# *In Vitro* Anthelminthic Efficacy of *Hypoestes forskaolii* (Vahl) R.Br (Acanthaceae) Extracts on Gastrointestinal Nematodes of Sheep

**DOI:** 10.3390/vetsci5040089

**Published:** 2018-10-16

**Authors:** Massimiliano D’Ambola, Antonio Bosco, Andrea Ariano, Laura Rinaldi, Ammar Bader, Alessandra Amadesi, Giuseppe Cringoli, Lorella Severino

**Affiliations:** 1 Department of Veterinary Medicine and Animal Productions, University of Naples Federico II, 80137 Naples, Italy; massimiliano.dambola@unina.it or mdambola@unisa.it (M.D.); boscoant@tiscali.it (A.B.); andrea.ariano@unina.it (A.A.); lrinaldi@unina.it (L.R.); alessandra.amadesi@unina.it (A.A.); cringoli@unina.it (G.C.); 2 Department of Pharmacy, University of Salerno, 84084 Fisciano, Italy; 3 Department of Pharmacognosy, Faculty of Pharmacy, Umm Al-Qura University, 21955 Makkah, Saudi Arabia; ammarosio@yahoo.it

**Keywords:** sheep, gastrointestinal nematodes, plant extracts, egg hatching assay, anthelminthic efficacy

## Abstract

The anthelminthic efficacy of the crude extracts of *Hypoestes forskaolii* (Vahl) R.Br (Acanthaceae) against gastrointestinal nematodes (GIN) in sheep was investigated using the in vitro egg hatch inhibition assay. Faecal samples were collected from sheep with naturally occurring infection of GIN (*Trichostrongylus* spp., *Chabertia ovina*, *Cooperia* spp., *Haemonchus contortus* and *Teladorsagia* spp.). Crude leaf extracts of *H. forskaolii* was obtained using increasing polarity solvents: *n*-hexane, chloroform, chloroform:methanol 9:1, methanol. Thiabendazole (0.2 µg/mL and 0.5 µg/mL) was used as a positive control and untreated GIN eggs in deionised water served as the negative control. All the extracts exhibited a weak ovicidal activity against GIN (less than 50% of egg hatch). Noteworthy, the *n*-hexane extract showed a percentage of inhibition of egg hatching greater than other extracts inhibiting the 30.8% at the concentration of 1 mg/mL showing a dose-dependent effect on nematode eggs hatching. Further studies are needed to investigate the effects of extracts used and to evaluate the ovicidal effects of other extracts of *H. forskaolii*.

## 1. Introduction

Infections by gastrointestinal nematodes (GIN) remain a major constraint to ruminants’ health, welfare and productive performance worldwide [1,2]. These parasites cause direct and indirect losses in different ways such as lowered fertility, reduced work capacity, reduction in food intake, low weight gain and low milk productions [3]. The administration of synthetic anthelminthics has long been considered the most effective way of controlling helminth infection to minimize losses caused by these parasites [4] but the use of these drugs has some disadvantages, such as development of anthelmintic resistance (AR), high cost, and risk of environmental pollution [5].

The availability and affordability of systemic anthelminthics to small-scale sheep farmers is a major problem in many developing countries [6]. This problem justifies the need for alternative control methods, such as the use of traditional medicinal plants, that are being examined in different parts of the world [7]. The screening and proper evaluation of medicinal plants could offer a possible alternative that may both be sustainable and environmentally acceptable [8].

Nowadays, about 50% of drugs used in modern medicine are of plant origin so the universal role of plants in the treatment of diseases is established by their employment in all important systems of medicine [9,10].

Anthelmintics derived from plants used for the treatment of parasitic infections in human and animals can offer an alternative to minimize some of these problems [11]. One of the novel approaches investigated is the use of indigenous plant preparations commonly used in the herbolary against human parasites. The benefit of using these as possible livestock dewormers is that they are non-toxic and inexpensive, features that are important for farmers in developing countries [12]. These possible ethnoveterinary alternatives would be viable for small scale livestock farmers who cannot afford the allopathic drugs and/or for larger, conventional farmers who cannot rely on the use of conventional veterinary products in their flocks [13]. Furthermore, plant-derived anthelminthic products are advantageous as they are less toxic, biodegradable, and environmentally friendly [14].

Species of the genus *Hypoestes* are used for chest and heart diseases, gonorrhea, cancer, liver protection and as antipyretic and antiphlogistic [15,16]. 

Various biological properties have been attributed, including antiplasmodial, antifungal, antileishmanial, antrypanosomal and cytotoxic properties [15,17]. *Hypoestes forskaolii* (Acanthaceae) is an herbaceous plant growing in Saudi Arabia: Their anthelmintic activity has not been tested so far. 

*Hypoestes forskaolii* is distributed in the Arabian Peninsula and many regions of Africa. Previous studies showed that this plant has an anti-parasitic effect although the leaves are not grazed by sheep. Furthermore, local shepherds use the leaves decoction to externally wash the sheep in order to kill insects and parasites, and the poultice of fresh plant is added to milk to poison house flies [18,19].

Some plants with anthelmintic activity are well known for their high toxicity e.g., *Consolida regalis* Gray (Ranunculaceae), *Calotropis procera* Dryand (Aiton) and *Rauvolfia vomitoria* Afzel (Apocynaceae) [20,21,22]. In addition, many plants with anthelmintic activity are not palatable for animals so they are rarely grazed by livestock e.g., *Dryopteris filix-mas* (L.) Schott (Dryopteridaceae), *Allium sativum* L. (Alliaceae), *Juglans regia* L. (Juglandaceae) and also *Consolida regalis* Gray (Ranunculaceae) [22].

Therefore, the aim of this study was to investigate the in vitro anthelmintic activity of the leaves (*n*-hexane, chloroform, chloroform:methanol 9:1, methanol crude extracts) of *H. forskaolii* on the eggs of GIN infected sheep in south Italy. A study conducted by Rinaldi et al. [23] shows that AR is rare in Italy, therefore, a suitable setting to study ovicidal and larvicidal properties of a plant extract.

## 2. Materials and Methods 

### 2.1. Plant Material

The aerial parts of *Hypoestes forskaolii* (Vahl) R.Br. were collected in Wadi Thee Ghazal, Taif in Saudi Arabia, in September 2013. The plant was identified by Dr. Ammar Bader. A plant sample (No. SA-EN 2013-2) is deposited in the herbarium of the laboratory of Pharmacognosy Faculty of Pharmacy at Umm Al-Qura University, Saudi Arabia.

### 2.2. Plant Extracts

The aerial parts (505.0 g), dried and pulverized, were subjected to maceration with increasing polarity solvents: *n*-hexane, chloroform (CHCl_3_), chloroform:methanol 9:1 (CHCl_3_:CH_3_OH) and methanol (CH_3_OH).The whole extraction process was three weeks long, during which the solvent was constantly renewed (3X2L). The residues obtained with the different solvents were filtered and evaporated under reduced pressure with a bath heated to temperatures less than 40 °C thus obtaining the four residues: *n*-hexane (25.8 g), chloroform (24.1 g), chloroform:methanol 9:1 (18.4 g), methanol (21.2 g).

Furthermore, all the extracts were analyzed using TLC (Thin Layer Chromatography) performed on precoated Kieselgel 60 F254 plates (Merck, Kenilworth, NJ, USA), 0.25 mm thick, with glass or aluminum as support. The spots were revealed using UV detection with a lamp at 254 or 366 nm and successively using specific spray reagents, allowing to the development of typical coloring that can give information about the nature of examined compounds. Ce_2_SO_4_/H_2_O_4_ and Dragendorff’s reagent were used. As eluting solvents in the TLC analyses, two mixtures of solvents were mainly used: CHCl_3_-MeOH-H_2_O (80:18:2) for apolar mixtures and n-BuOH-AcOH-H_2_O (60:15:25) for polar mixtures.

To compare the activity of these extracts of *H. forskaolii*, five final concentrations in wells were tested: 1 mg/mL, 0.5 mg/mL, 0.25 mg/mL, 0.05 mg/mL, 0.005 mg/mL. To increase the solubility of the crude extracts a solutions of dimethyl-sulfoxide (DMSO)/H_2_O 0.5% were used for methanolic extract and Tween 85/H_2_O 5% were used for *n*-hexane, chloroform, chloroform:methanol 9:1 extracts.

### 2.3. Recovery of GIN Eggs

Fresh fecal samples containing GIN eggs were obtained from naturally infected sheep and processed within 4 h of collection. Specifically, GIN eggs were extracted from the positive samples using the mass recovery method, i.e., a method that employs 5 sieves of different dimensions (1 mm, 250 µm, 212 µm, 63 µm and 38 µm) in order to separate the eggs from the feces [24]. The latter filter (38 µm) was washed and eggs were suspended in deionised water and then visualized under a microscope (Leica, Wetzlar, Germany, 20×) to record if embryonation had not begun. Ten aliquots of 0.1 mL were taken, and the number of eggs counted [25]. The mean number of eggs counted in these aliquots was 150 per 0.1 mL of egg suspension.

### 2.4. Fecal Cultures

In order to identify the GIN genera involved in this study, larval cultures were needed. An aliquote of fecal positive sample containing GIN eggs was broken up finely, using either a large pestle and mortar or spatula and were placed in a glass jar or petri dishes which was closed and incubated at a temperature of about 25 °C for 10 days. After incubation, samples were examined for larvae using a binocular microscope [26]. Third stage larvae were identified using the morphological keys proposed by Van Wyk and Mayhew [27]. When a coproculture had 100 or less third stage larvae, all were identified; when a coproculture had more than 100 larvae, only 100 were identified [28].

### 2.5. Egg Hatch Assay (EHA)

The EHA procedure followed that recommended by the World Association for the Advancement of Veterinary Parasitology (WAAVP) [29]. The protocol to prepare thiabendazole (TBZ, Sigma, Saint Louis, MO, USA) solution used deionised water with a neutral pH. Thus, for the preparation of solution A, 50 mg TBZ were dissolved in 5 mL water. Subsequent dilutions were made in deionised water [30]. Stock solution B (1 mg TBZ per mL) was obtained by adding 1 mL of stock solution A (10 mg TBZ per mL) to 9 mL water, then 400 µL and 1000 µL of stock solution B were added to 9.6 mL and 9 mL of water, respectively. Therefore, the final concentrations of TBZ in wells were: 0.2 and 0.5 µg per mL. In our study, TBZ was used as a positive control.

Five working concentrations were prepared of each *H. forskaolii* extracts: 1 mg/mL, 0.5 mg/mL, 0.1 mg/mL and 0.01 mg/mL. Egg suspension (100 µL with 150 eggs) was placed in plastic wells (24 well tissue culture test plates), 900 µL of water was added and finally 1 mL of each extract concentration was added. Therefore, the final concentrations in wells were: 1 mg/mL, 0.5 mg/mL, 0.25 mg/mL, 0.05 mg/mL and 0.005 mg/mL. Each sample was tested in triplicate and at least two negative control samples were used (including the sample in deionised water without any drug/plant extracts). The plates were incubated for 48 h at 25–27 °C and the assay stopped by adding two drops of Lugol’s iodine. The eggs and larvae in each well were carefully washed in a petri dish marked with a grid and counted using a binocular microscope [24]. Thereafter, the number of larvae present per well was counted, and the percentage hatched was determined as the ratio between the number of larvae to the number of eggs deposited per well. A mean percentage of hatching was calculated for each concentration of each plant extracts. 

### 2.6. pH Analysis

The pH of each extract was measured using a pH meter (Hanna instruments Hi-223 calibration check microprocessor pH meter). All pH readings were conducted on every working solution in triplicate.

### 2.7. Statistical Analysis

Statistical analyses were performed using Graphpad Prism 5 to calculate values for different extract treatments. In particular, for each extract of *H. forskaolii* Standard Deviation (SD) was calculated, that reflects the variation in normally distributed data. Differences between crude extracts of *H. forskaolii* activity were analyzed using a one-way ANOVA followed by a Tukey’s multiple comparison test. Furthermore, a One-Sample *t*-test was used to compare % eggs found in each extract to the control mean.

## 3. Results

### 3.1. Coproculture

The most prevalent genera of GIN were *Trichostrongylus* spp. (42.6%), followed by *Chabertia ovina* (22.9%), *Cooperia* spp. (21.3%), *Haemonchus contortus* (8.1%) and *Teladorsagia* spp. (5%).

### 3.2. EHA Test

The results of the EHA for the four extracts of *H. forskaolii*, TBZ and their water controls are shown in Table 1. The results showed a mean of 7.1% of eggs in the negative control (deionised water), a mean of 96.9% of eggs in the first positive control (0.2 µg/mL of TBZ) and a mean of 97.2% of eggs in the second positive control (0.5 µg/mL TBZ). Extract pH analysis did not show significant changes in the variations of acidity or basicity. The measured pH did not deviate from neutrality, showing a range between 6.8 and 7.2. 

Overall, significant differences were observed in methanolic (*p* < 0.005), chloroform (*p* < 0.005) and *n*-hexane (*p* < 0.001) extracts of *H. forskaolii* respect to the control. In particular, Table 1 shows that the *n*-hexane extract has a percentage of inhibition of egg hatching greater (*p* < 0.05) than other extracts inhibiting the 30.8% at the concentration of 1 mg/mL showing a dose-dependent effect on GIN eggs hatching. The chloroform:methanol (9:1) extract of *H. forskaolii* showed no significant difference in respect to the control and showed an high turbidity at 1 mg/mL concentration, therefore it was impossible to examine the sample with a binocular microscope. Due to high turbidity, the SD of the chloroform:methanol (9:1) extract is higher than the SD of other *H. forskaolii* extracts (*p* < 0.05). 

## 4. Discussion

GIN are a major cause of disease in grazing sheep. Despite the progress in the development of parasite vaccines, anthelmintics are still indispensable for worm treatment and control [31]. However, the indiscriminate use of anthelmintics to control helminths has led to wide AR in sheep [32]. The development of AR to commercially available drugs, as well as the risks that are associated with the presence of these products in the environment, and in foods of animal origin, have encouraged the search for new active ingredients that are less toxic, able to minimize the presence of drug residues in food of animal origin and more efficient. 

In this context, products of plant origin may be an effective alternative for the control of parasites [33]. The use of plants with anthelmintic properties is considered as one of the most viable alternative methods for the control of GINs, even though crude drugs are less efficient with respect to cure of diseases but are relatively free from side effects.

A large number of medicinal plants could possess anthelmintic properties in the traditional system of medicine and are also used by ethnic groups in the world. Unfortunately, only a small number of plant species shows an appreciable ovicidal activity [34].

The results of this study show that methanolic, chloroform and *n*-hexane extracts from the leaves of *H. forskaolii* exhibit an ovicidal activity against GIN, whereas only two works have succeeded in demonstrating that two plants, belonging to Acanthaceae family, show a considerable in vitro ovicidal activity, but only within their polar residues, such as aqueous and ethanolic. Nevertheless, there are no studies which focus on apolar extracts such as the *n*-hexane and the chloroformic. In a study by Al-Shaibani et al. [35], the aqueous and ethanolic extracts of *Adhatoda vasica* presented ovicidal activity against the GIN eggs. The ethanolic extract was slightly more effective compared to aqueous extract on eggs. In the study of Adamu et al. [6] it was instead considered only the aqueous extract of *Acanthus montanus* (Nees) T. Anders (Acanthaceae). In this work, in addition to polar methanolic extract, the apolar and medium polar residues, respectively the *n*-hexane, chloroform and chloroform:methanol 9:1, were analyzed for the first time in a plant belonging to the Acanthaceae family.

Moreover, considering that this study aims to analyze the biological activity of *H. forskaolii* polar and apolar residues, the ovicidal action can be given by the presence of different bioactive molecules. Previous phytochemical investigations carried out on various *Hypoestes* species revealed the existence of different classes of phytochemicals, as diterpenoids (labdane-, furanolabadane-, fusicoccane-, isopimarane-, verticillane- and dolabellane-types), alkaloids, lignans, and pentacyclic triterpenes [36]. Regarding *H. forskaolii*, the presence of terpenoids, particularly the fusiccocane type, has already been mentioned in the literature [37] while no scientific reports have shown the presence of alkaloids in this species. Absence of alkaloids in *H. forskaolii* was further confirmed by TLC analysis performed with the specific high-selective Dragendorff reagent for this class of molecules.

In conclusion, in the adopted experimental conditions, the *n*-hexane extract showed a rather mild egg hatch inhibition and a dose-dependent effect on nematode eggs hatching while on the contrary in other studies, conducted in various experimental conditions, the *n*-hexane extract and other apolar extracts from different plant species showed dissimilar ovicidal activities against nematodes in sheep, such as *H. contortus*, *Teladorsagia* spp., *Trichostrongylus* spp., *Strongyloides papillosus*, *Oesophagostomum columbianum*, and *Chabertia ovina* [38,39,40].

According to the adopted experimental approach, it was not possible to test the extracts in concentrations higher than 1 mg/mL because of the turbidity of the solutions created in wells during the EHA. Despite using surfactant substances to increase the solubility of the extracts, it was impossible to count the eggs or larvae in those wells where concentrations exceeded the highest cited. Moreover, limited to the chloroform:methanol 9:1 extract, it was impossible to analyze the concentration of 1 mg/mL due to the turbidity of the solution that was already created at that concentration.

## 5. Conclusions

The inhibition of egg hatch in vitro is an indication of the possible usefulness of various plant as potential anthelminthic and, in particular, *H. forskaolii* appears to possess a dose-dependent anthelmintic activity with a mild activity shown. For these reasons, under our experimental conditions we have obtained encouraging results and extracts of this plant can be tested on other species of helminths and it could be interesting to evaluate the efficacy of *n*-hexane extracts of the other plants of Acanthaceae family in order to study their activity, toxicity, mechanism of action and identification of phytochemicals. The present study must be considered a preliminary attempt to evaluate the ovicidal action of crude leaf extracts of *H. forskaolii* and further studies are necessary to provide detailed information on the mode of action and chemical nature of any specific nematode ova inhibitory compounds identified. 

## Figures and Tables

**Table 1:** Percentages of gastrointestinal nematode (GIN) eggs and egg hatch after treatment at various concentrations with thiobendazole, leaf *n*-hexane, chloroformic, chloroformic:methanolic (9:1) and methanolic extract of *H. forskaolii* and deionised water.

	*n*-Hexane (mg/mL)		Chloroformic		Chloroformic:Methanolic (9:1) (mg/mL)		Methanolic	
Concentration (mg/mL)	% Egg ± SD	% Egg Hatch ± SD	% Egg ± SD	% Egg Hatch ± SD	% Egg ± SD	% Egg Hatch ± SD	% Egg ± SD	% Egg Hatch ± SD
0.005 mg/mL	15.00 ± 0.68	85.00 ± 0.68	10.00 ± 0.20	90.00 ± 0.20	8.25 ± 2.05	91.75 ± 2.05	0.67 ± 0.58	99.33 ± 0.58
0.05 mg/mL	17.82 ± 0.65	82.18 ± 0.65	10.19 ± 0.19	89.81 ± 0.19	2.78 ± 3.08	97.22 ± 3.08	1.00 ± 1.00	99.00 ± 1.00
0.25 mg/mL	18.81 ± 0.24	81.19 ± 0.24	6.70 ± 1.08	93.30 ± 1.08	4.48 ± 2.56	95.52 ± 2.56	5.67 ± 0.58	94.33 ± 0.58
0.5 mg/mL	20.00 ± 0.79	80.00 ± 0.79	9.77 ± 5.14	90.23 ± 5.14	8.09 ± 4.51	91.91 ± 4.51	5.33 ± 1.15	94.67 ± 1.15
1 mg/mL	30.81 ± 0.83	69.19 ± 0.83	7.34 ± 0.36	92.66 ± 0.36	ND *	ND *	10.67 ± 0.58	89.33 ± 0.58
	**TBZ (µg/mL)**							
	% Egg ± SD	% Egg Hatch ± SD	% Egg ± SD	% Egg hatch ± SD	% Egg ± SD	% Egg Hatch ± SD	% Egg ± SD	% Egg Hatch ± SD
0.2 mg/mL	95.00 ± 1.00	5.00 ± 1.00	99.50 ± 0.50	0.50 ± 0.50	95.60 ± 5.50	4.40 ± 5.50	97.33 ± 2.52	2.67 ± 2.52
0.5 mg/mL	95.88 ± 0.03	4.12 ± 0.03	99.50 ± 0.50	0.50 ± 0.50	96.53 ± 2.42	3.47 ± 2.42	97.00 ± 3.00	3.00 ± 3.00
	**DMSO/Tween 85/Deionised Water Control**							
	% Egg ± SD	% Egg Hatch ± SD	% Egg ± SD	% Egg Hatch ± SD	% Egg ± SD	% Egg Hatch ± SD	% Egg ± SD	% Egg Hatch ± SD
	3.67 ± 1.53	93.33 ± 1.53	17.50 ± 3.32	82.50 ± 3.32	4.50 ± 0.50	95.50 ± 0.50	2.67 ± 2.08	97.33 ± 2.08

* ND: The turbidity of the solution did not allow the identification of GIN eggs; % Egg: Percentage of eggs found in each plate; % Egg Hatch: Percentage of larvae found in each plate; SD: Standard Deviation.
